# Results of community-based TB and HIV screening among transgender women and male sex workers in Pakistan

**DOI:** 10.1371/journal.pgph.0000913

**Published:** 2023-01-11

**Authors:** Sharaf Ali Shah, Shahina Qayyum, Saifullah Baig, Nikhat Iftikhar, Rubab Lubna Bukhari, Wajid Ali, Marina Smelyanskaya, Jacob Creswell

**Affiliations:** 1 Bridge Consultants Foundation, Karachi, Pakistan; 2 Dow University of Health Sciences, Karachi, Pakistan; 3 MDG Achieving Organization, Karachi, Pakistan; 4 Pireh Mala Health Society, Larkana, Pakistan; 5 Innovations & Grants Team, Stop TB Partnership, Geneva, Switzerland; Boston University, UNITED STATES

## Abstract

In Pakistan and globally, a large proportion of people with TB who are not receiving treatment are key populations with poor access to diagnosis and care. Transgender women and male sex workers (MSW) are heavily stigmatized and marginalized groups. While HIV rates are well documented among these key populations, little such data exists for TB. We engaged local organizations working with transgender women and MSW communities in Karachi and five urban cities in Sindh Province. People from the communities served as screening facilitators and treatment supporters. Verbal screening was followed by testing with Xpert MTB/RIF and HIV testing was offered. People with TB were supported through treatment. We screened 18,272 transgender women and 24,253 MSW. 8,921 (21.0%) individuals had presumptive TB and 7,472 (83.8%) provided sputum samples. We detected 438 (5.9%) people with positive results including 140 transgender women and 298 MSW. Including people diagnosed clinically, 625 people with TB were identified and 98.1% initiated treatment. Overall, 1.5% of people screened had TB, 1.7% among MSW and 1.1% among transgender women. Of 1,508 people tested for HIV, 243 had HIV infection (HIV+). The rates of TB among HIV+ transgender women (8.8%) were slightly lower than among MSW (10.3%). Previously, few attempts have been made to address TB in transgender women and MSW. Our work shows that these groups carry a significant burden of both TB and HIV in Pakistan and do not regularly access services. Effective interventions should include the engagement of community leaders and peers.

## Introduction

Universal health coverage is a target under Sustainable Development Goal Three that prioritizes better health and wellbeing. This proven strategy stops unnecessary deaths and increases health and development outcomes [1]. Globally, tuberculosis (TB) is a leading killer among infectious diseases, primarily because millions of people with TB are not diagnosed [2]. Pakistan, ranking fifth among high burden countries, accounts for 8% of the global gap in TB treatment coverage (the difference between estimated incidence and TB notifications) [2]. Challenges faced by Pakistan in fighting TB include an unregulated private sector where many people seek care for TB [3] and lack of focused social support programs for populations at the highest risk for TB [4]. To end TB, these challenges must be tackled, and countries must implement effective strategies for universal health coverage, that include community engagement and a focus on the most vulnerable [5].

As defined in the Global Plan to End TB, TB key populations have increased exposure to TB, limited access to TB care, or/and increased risk of developing TB. Importantly, key populations will differ by country and setting [6]. Key populations often include people who are incarcerated [7], substance abusers [8, 9], miners [10], and people living with HIV (PLHIV) [11]. Globally, HIV infection is the strongest risk factor for developing TB [12]. In Pakistan, HIV prevalence in the general population is low at 0.1% [13] and HIV rates are also low among people with TB [14]. However, a recent Integrated Biological and Behavioral Survey (IBBS) covering four provinces documented high rates of HIV among people who inject drugs (38.4%), transgender women (7.1%), and male sex workers (MSW) (5.3%) [15]. The TB burden among these populations has not been documented or compared.

The Transgender Persons (Protection of Rights) Act [16] passed by Pakistan’s legislators in 2018 defines a transgender individual as “a transgender man, transgender woman, Khawaja Sira or any person whose gender identity or gender expression differs from the social norms and cultural expectations based on the sex they were assigned at the time of their birth.” Transgender women, *hijra*, or *Kawaja Sira* as they are called in Pakistan, are widely discriminated against, have low access to education and employment, live on the margins of society and often engage in sex work to earn money [17, 18]. Ethnographic studies and programmatic experience with transgender communities in South Asia show that transgender people leave their homes at a young age [19, 20]. They can be abandoned by their families due to their gender identity and often live in close-knit hierarchical communities led by gurus (teachers). Gurus are older, more experienced members of the transgender community and provide shelter, food, a supportive environment, protection, and other necessities to their younger counterparts. Gurus often play a parental role so, as per family tradition in Pakistan, younger transgender women in turn share their income with the Gurus.

In Pakistan same-sex behavior and intercourse are still criminalized as is transactional sex, putting MSW on the margins of society [21]. MSW in Pakistan engage in selling sex to men because of financial need, or various local cultural practices [22]. Most of them are selling on streets, or in venues, rather than more discreet online work. Compared to transgender women, MSW in Pakistan are younger and more likely to use injection drugs or have a partner who injects. They experience discrimination, physical abuse and sexual violence at a higher rate than men who have sex with men but are not involved in sex work [15].

While working to ensure all people with TB receive diagnosis and care, countries should adhere to the principles of human rights [6]. Successful community-based intervention require an understanding of the community dynamics, existing community structures, traditions, and behavioral patterns [23]. With support from the Global Fund to fight AIDS, TB and malaria, Pakistan has committed to conducting legal and gender assessments which document barriers that vulnerable TB-affected populations face in accessing TB care to inform a national response and key populations require special focus [24]. Bridge Consultants Foundation, a non-governmental organization (NGO) in Pakistan, has been focusing on the health of the most marginalized populations. To help address the lack of focus on TB among transgender women and MSW, the stigma and HIV risk these groups face, document potential differences of TB burden in the populations, and support Pakistan’s efforts to both address transgender rights and advance the TB response, we describe the results of an intervention to measure the burden of TB among these groups, and expand access to TB diagnosis and treatment through peer outreach and community involvement.

## Materials and methods

The interventions were implemented in Karachi, the most populated city of Pakistan, and five other districts of Sindh province including, Hyderabad, Mirpurkhas, Benazirabad, Sukkur and Larkana from May 2017 to August 2018. These districts were selected based on mapping results of previous IBBS surveys indicating transgender women’s residences and gathering areas of MSW.

Twelve public sector basic management units (BMUs which are TB reporting sites), six in Karachi, two in Hyderabad, and one each in the four other cities were selected based on accessibility by public transport and availability of rapid molecular testing with a WHO recommended molecular diagnostic test, Xpert MTB/RIF (Xpert) (Cepheid Inc., Sunnyvale, CA, USA). Staff at the selected facilities (doctors, paramedics, and lab technicians) were trained to better understand the sensitivities of providing care to transgender women and MSW, and intervention protocols.

### Obtaining community buy-in and engagement

Due to the marginalization of MSW (all of whom were men who have sex with men) and transgender women, outreach efforts had to be coordinated with the respective communities. Therefore, ensuring trust and facilitating close linkages in the community was crucial. MSW in Pakistan have weak social support networks [15, 17] and can be harder to reach and engage than other at risk populations. We engaged local NGO and community-based organizations (CBO) working with transgender women and MSW to approach their leaders and utilized our own contacts within these communities. Individuals from the communities were engaged as treatment supporters as part of the intervention.

### Patient and public involvement

Individual leaders from both transgender and MSW communities were included at the design of the intervention and continued to work in the implementation and results dissemination as part of ongoing engagement through work on HIV activities. As part of the trainings, eight outreach workers (ORW) were employed and trained in verbal screening to identify people with presumptive TB. ORW were also trained on safe collection of sputum, transport the samples to laboratory, collecting results, and communicate results to people with presumptive TB. Fifty treatment supporters from the MSW and transgender communities were trained on monitoring treatment, filling treatment card, and preventing loss to follow up. Leaders MSW and transgender communities were helped map outreach activities, and design messages for providing support for people identified with TB symptoms, TB disease and/or HIV.

### Community-based case finding approach

Using the mapping from the IBBS survey, ORW, facilitated by the Gurus and focal persons, approached transgender women and MSW at their residences, workplaces, and gathering areas. After informed verbal consent, individuals were asked if they had been previously diagnosed with TB and if so, if it was within the last 12 months, to assess health seeking behavior related to TB diagnosis and treatment.

All individuals were then verbally screened for the presence of any of the following symptoms: prolonged cough (at least two weeks), any cough, fever, night sweats, or weight loss. Those who answered yes to prolonged cough or any cough plus any other symptom were considered to have presumptive TB and a sputum specimen was requested. Individuals who could not produce a sample were requested to provide a morning specimen. Samples were collected by the ORW the following morning and transported to designated BMU labs for testing using Xpert.

**Fig 1:**
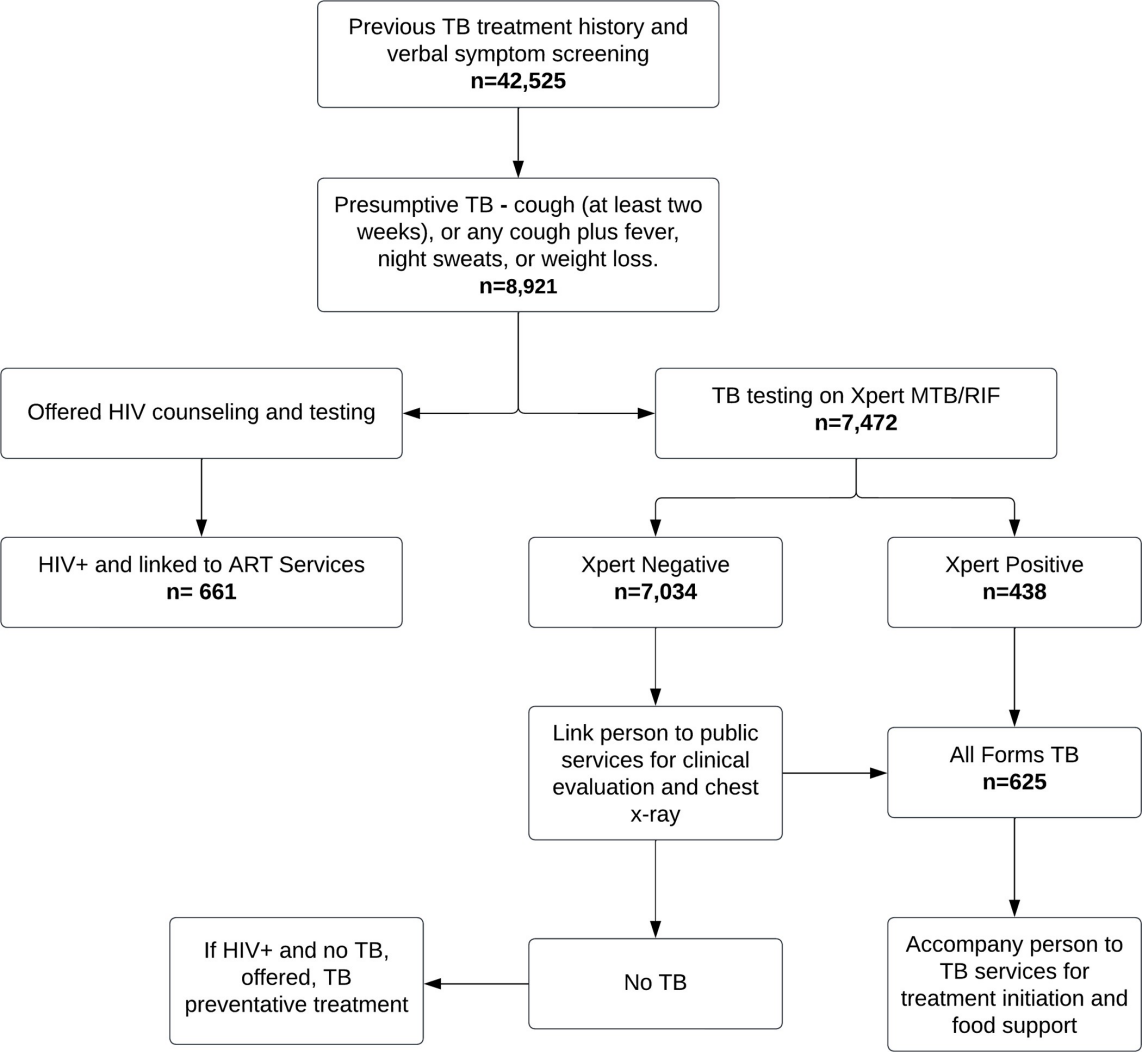
Screening approach and treatment pathway for community TB and HIV screening. TB = Tuberculosis, ART = antiretroviral therapy HIV = Human Immunodeficiency Virus.

ORW collected the Xpert results and communicated them along with health education and counseling. They accompanied individuals with Xpert-positive TB (MTB+) to the BMU for treatment registration and provided support in navigating the health services. People whose Xpert results were negative were examined at BMU including chest X-ray (CXR) for possible clinical diagnosis of TB. The treatment supporters followed up people initiated on TB treatment until the completion of treatment and maintained their treatment records (Fig 1).

All individuals with presumptive TB were also asked about their HIV status. Those who did not know their HIV status were offered HIV testing with pre-test and post-test counseling according to national guidelines. Those who consented were tested for HIV. All people with HIV (already known and newly identified) were linked to care, support, and treatment services through community home based care (CHBC) and antiretroviral therapy (ART) programmes.

ORW followed all people receiving TB treatment monthly, providing them with a monthly nutritional support package consisting of basic food items (rice, wheat flour, cooking oil, lentils, sugar, tea, and powdered milk) for the duration of the treatment. ORW coordinated with treatment supporters through telephone for side effect monitoring. Treatment supporters were also provided direct links to the health facilities.

Progress reports from the intervention were presented in quarterly review meetings participated by different stakeholders including National and Provincial TB Programs, Stop TB Partnership Pakistan, project CBOs and NGOs, and local health departments. These were chaired by the Director General Health Services, Sindh.

### Data analysis

Data for people with presumptive TB was collected on study registers and entered in Microsoft Excel. Data for registered cases was abstracted from the individual’s TB card (form TB 01) and official TB register (TB 03) at the BMU. Simple counts and proportions were calculated throughout the TB and HIV screening cascade. We compared differences between transgender women and MSW using chi-squared tests. Official program data from the BMU’s TB 03 was used for treatment outcomes. Analysis was done using Microsoft Excel.

### Ethics

The Institutional Review Board of Bridge Consultants Foundation reviewed and approved the study. Oral consent was approved and used due to low literacy levels of the populations engaged and documented in the ORW screening forms.

## Results

### TB screening

From 20 May 2017 to 30 April 2018 a total of 42,525 people (18,272 transgender women and 24,253 MSW) were screened for TB symptoms (Table 1). Overall, 98 individuals or 0.2% (41 transgender women and 57 MSW) reported previous TB treatment. Among them, 23 were diagnosed in the previous 12 months. Among all people verbally screened, 8,921 (21.0%) had presumptive TB, and sputum samples from 7,472 (83.8%) of them were collected and tested using Xpert. The 1,449 people whose sputum was not collected could only produce a salivary specimen. The proportions of transgender women and MSW screened who had presumptive TB were similar (3,819 (20.9%), and 5,102 (21%) respectively) as were the proportions who were able to produce sputum, 83.4%, and 84%, respectively.

**Table 1:** TB screening cascade among transgender women and male sex workers.

	Transgender n (%)	MSW n (%)	Total n (%)	Chi square p value
People screened	18,272	24,253	42,525	
Presumptive TB (% of screened)	3,819 (20.9)	5,102 (21.0)	8,921 (21.0)	0.79
Tested for TB (% of presumptive)	3,184 (83.4)	4,288 (84.0)	7,472 (83.8)	0.41
Confirmed B+ detected (% of tested)	140 (4.4)	298 (6.9)	438 (5.9)	<0.001
People clinically evaluated (% of tested negative)	955 (31.4)	1730 (43.4)	2,685 (38.2)	<0.001
People clinically diagnosed (% of evaluated)	62 (6.5)	125 (7.2)	187 (7.0)	0.51
All forms TB (% of presumptive)	202 (5.3)	423 (8.3)	625 (7.0)	<0.001
MTB+ starting treatment (% of diagnosed)	136 (97.1)	293 (98.3)	429 (97.9)	0.65
All forms TB starting treatment (% of diagnosed)	197 (97.5)	416 (98.3)	613 (98.0)	0.7
Number Needed to Screen (All Forms)	90	57	68	

MSW = Male Sex Workers, TB = Tuberculosis, AF = All forms, MTB+ = Bacteriologically confirmed TB

Among 7,472 people tested by Xpert, 438 (5.9%) had MTB+ results including 140 transgender women and 298 MSW. Transgender women had significantly lower positivity rates (4.4%) than MSW (6.9%) (p<0.001). Of the 7,034 people with Xpert negative (MTB-) results who were referred to the BMUs for further investigation we could track 2,685 (955 transgender women and 1,730 MSW). Clinical investigation and x-ray screening resulted in an additional 187 people with clinically diagnosed TB. In total, we identified 625 people with TB. Six people (1.4% of those with MTB+ results) had rifampicin resistance. Of these, one was lost to follow up, one died, and four started second line treatment. The number needed to screen (NNS) to identify a person with TB was 68 overall while it was higher for transgender women compared to MSW (90 and 57).

### HIV screening and testing

Among the 8,921 people with presumptive TB, 44.8% reported knowing their HIV status including 1,582 (41.4%) transgender women and 2,417 (47.4%) MSW (Table 2). Overall, 418 (4.7%) reported they knew they had HIV infection. The percentage of MSWs living with HIV (14.2%) was higher than that of transgender women (4.7%) (p<0.001). Among people with presumptive TB, 3,581 (40.1%) self-reported as HIV negative.

**Table 2:** HIV screening and testing among those identified as presumptive for TB, Sindh Pakistan.

	Transgender n (%)	MSW n (%)	Total n (%)	Chi Square *p value*
**People with presumptive TB**	3,819	5,102	8,921	** **
**HIV status known**	1,582 (41.4)	2,417 (47.4)	3,999 (44.8)	<0.001
**HIV status not known**	2,237 (58.6)	2,685 (52.6)	4,922 (55.2)	
**Among those with known HIV status**	** n = 1,582**	** **		
**HIV -**	1,508 (95.3)	2,073 (85.8)	3,581 (89.5)	<0.001
**HIV +**	74 (4.7)	344 (14.2)	418 (10.5)	
**Among those with unknown HIV status**	**n = 2,237 **			
**HIV tested (%)**	392 (17.5)	1,116 (41.6)	1,508 (30.6)	<0.001
**HIV+ (% of tested)**	74 (18.9)	169 (15.1)	243 (16.1)	0.98
**Total HIV + (% of presumptive TB)**	148 (3.9)	513 (10.1)	661 (7.4)	<0.001
**TB diagnosed (% among all HIV+)**	13 (8.8)	53 (10.3)	66 (10.0)	0.74
**Initiating TB treatment (% of diagnosed)**	11 (84.6)	53 (100)	64 (97.0)	0.05
**Number needed to screen among HIV+**	11	10	10	

MSW = Male sex workers, HIV = Human Immunodeficiency Virus, TB = Tuberculosis

Among those who did not know their status, 1,508 (30.6%) consented to HIV testing. Acceptance among transgender women for HIV testing was less than MSW (17.5% compared to 41.6%, p<0.001). Among the 1,508 individuals tested for HIV, 243 (74 transgender women and 169 MSW) were newly identified as HIV+ and rates were similar between the two groups. All were linked with ART services through the Provincial AIDS Control Program. Thus 661 people were identified as people living with HIV (PLWHIV) (148 transgender women and 513 MSW). Sputum was tested from 643 (97.3%) of PLHIV (142 transgender women and 501 MSW). TB was detected in 66 (10%). The rates of TB among HIV+ transgender women (8.8%) were slightly lower than among MSW (10.3%) but statistically insignificant. All but two of the PLWHIV with TB registered for ART and initiated anti- TB treatment. Among PLWHIV, the NNS were similar, 11 for transgender woman and 10 for MSW.

### Treatment outcomes

Of the 625 people diagnosed with TB, 613 (98%) were registered for treatment. Among the 12 people (1.9%) with pre-treatment loss to follow-up, three died before treatment, and nine refused to be treated (Table 3). Among the 613 who initiated anti-TB treatment, 545 (88.9%) were either cured or completed treatment successfully, while 19 died and 28 were lost to follow-up. Overall, treatment success rate was similar among transgender women and MSW (87.8% and 89.4% respectively). Among the 64 PLWHIV initiating treatment, the rate of treatment success was lower (62.5%) while loss to follow-up (18.5%) and death rates (14.1%) were higher. Of those who died, 47% (9 of 19) were PLWHIV.

**Table 3:** TB treatment outcomes for transgender women and male sex workers in Sindh, Pakistan.

	Transgender Women			MSW			Total
	TB All Forms			TB All Forms			
	HIV-	HIV+	ALL	HIV-	HIV+	ALL	
Registered	186	11	197	363	53	416	613
Cured	79	2	81	169	5	174	255
Treatment completed	90	2	92	167	31	198	290
Treatment failure	0	0	0	2	0	2	2
Died	4	3	7	6	6	12	19
LTFU	7	2	9	9	10	19	28
Not evaluated	4	1	5	4	0	4	9
Transferred out	1	1	2	0	1	1	3
% negative treatment outcome	5.9%	45.5%	8.1%	4.7%	30.2%	7.9%	8.0%
% treatment success	90.9%	36.4%	87.8%	92.6%	67.9%	89.4%	88.9%

MSW = Male sex worker, LTFU = Loss to follow up, HIV+ = People living with HIV

## Discussion

Our study is first to address the burden of TB in transgender women and MSW in Pakistan. Even outside Pakistan, few countries have sought to engage these groups in TB screening. We also demonstrated that through active case finding involving community outreach, access to HIV services for transgender women and MSW can be improved and the barriers these populations face in health care settings can be addressed. While the TB burden among transgender women and MSW in Sindh Pakistan is alarmingly high, it was not unexpected, considering high levels of HIV [25, 26], poverty, and marginalization [17] among these populations. Our findings are consistent with the scarce data that exists for these populations. The TB programme in India is one of the few countries to report TB among transgender populations and in 2020 notified TB in per 426/100,000 compared to 134/100,000 for women and 211/100,000 among men [27–29]. A small study in India identified TB in four of seven transgender people attending ART clinic [30]. MSW and transgender women were identified as a TB risk population in a study in Bangladesh [31].

In our non-representative sample, prevalence of TB among transgender women and MSW was 1.1% and 1.7% respectively. Overall, the death rate was high (3%), but 14% among PLHIV, suggesting delayed service provision and/or treatment barriers. Our findings demonstrate that only a small fraction of these populations had been previously diagnosed with TB and/or tested for HIV suggesting that while the burden of TB (and HIV) is high in transgender women and MSW of Pakistan, current health services are not meeting their needs.

The number needed to screen was lower among MSW than for transgender women (57 and 90 respectively) and 68 overall, and consistent with other population groups at higher risk of developing TB [32, 33] and indicates that large numbers of individuals with TB can be reached by screening transgender women and MSW in Pakistan if the communities can be engaged. While the NNS for PLWHIV was much lower at 10 overall, these individuals were only identified by conducting the outreach and most NNS studies in PLWHIV are facility-based. The numbers of people we screened using community outreach demonstrate the large numbers of marginalized people which may not be accounted for with standard approaches [15].

Pakistan has a public health infrastructure to reach a large portion of the population for TB care, but access to these basic services remains elusive for socially marginalized groups like transgender women and MSW. The MSW in our study were all MSM, a group that is often included in IBBS but may have even higher risk. Other studies have shown that despite available TB services, some groups will not access care for TB or other conditions, community- or peer-based outreach is the only way to provide care for these groups [34–36]. We demonstrate that many previously unreached people with TB can be diagnosed and effectively linked with care by involving members of these populations and giving them ownership over the intervention. Previous peer-based interventions for TB with key populations in Bangladesh [31], and Cambodia [37] demonstrated similarly positive results. This intervention, besides addressing the health needs of marginalized populations, also improved the ability of the public health system to serve transgender women and MSW, and increased trust of these groups into the public health sector through training and public-private collaboration. This is a dynamic necessary to make these interventions sustainable and foster positive behavioral change toward improved health care seeking [38–40].

While our intervention focused on TB case finding, it had an impact on expanding access to HIV testing and treatment as well. Only about 14% of Pakistanis living with HIV know their status [13]. While efforts to test all PLWHIV for TB are being scaled up globally, HIV testing is much lower in Pakistan [2]. TB programs could be more proactive, especially with key affected populations for HIV, and offer HIV testing and linkages to HIV services.

The main limitation to our study is non- representative sample as we accessed the community through leaders and Gurus, not through planned recruitment. Nevertheless, the high rates of TB documented should raise concerns for national and provincial leaders. We were also limited in providing clinical services to people with negative Xpert results. We confirmed 70% of cases with Xpert. In Pakistan overall, more than 50% of notified cases were bacteriologically unconfirmed in 2018 [2] and the high prevalence of HIV in our study increases the likelihood we missed people with TB and our results underestimate the TB burden.

Pakistan’s National Health Vision for 2016–2025 reinforces the country’s commitment to UHC [41] and so do the country’s recent commitments made during Global Fund grant negotiations [24]. These high-level commitments, along with the work at national and provincial levels to advance the rights of transgender women in Pakistan could bring greater access to health services. Others, however, whose activities are still considered criminal, will remain marginalized and can only be reached through community engagement. Pakistan’s path to UHC and TB and HIV elimination must include mobilization of communities, work with NGOs and CBOs, and a focus on those who are most marginalized.

## Conclusion

Our intervention is a first of its kind in Pakistan, and among the first globally. Previously, few attempts have been made to address TB in transgender women and MSW. Our work shows that these groups carry a significant burden of both TB and HIV in Pakistan, have low access to services and have high mortality. To address TB in these communities, engagement of community leaders and peer counselors is shown to be effective. Because of how embedded these communities are in Pakistani society, marginalizing these groups is harmful, and poses a challenge to ending TB in the country. We urge more focus on these and other key populations in Pakistan and globally as commitments to eliminate TB and achieve UHC are being made.
